# Postoperative Ratio of C-Reactive Protein to Albumin as a Predictive Marker in Patients with Crohn's Disease Undergoing Bowel Resection

**DOI:** 10.1155/2021/6629608

**Published:** 2021-02-26

**Authors:** Hangfen Zhao, Huaying Liu, Weilin Qi, Wei Liu, Lingna Ye, Qian Cao, Xiaolong Ge, Wei Zhou, Xianfa Wang

**Affiliations:** ^1^Department of General Surgery, Sir Run Run Shaw Hospital, School of Medicine, Zhejiang University, Hangzhou 310016, China; ^2^Department of Medicine, Guangxi Medical College, Nanning 530000, China; ^3^Department of Gastroenterology, Sir Run Run Shaw Hospital, School of Medicine, Zhejiang University, Hangzhou 310016, China

## Abstract

**Background:**

The ratio of C-reactive protein (CRP) to albumin (CAR) has a significant correlation with postoperative complications and acts as a predictor in patients with pancreatic cancer and colorectal cancer. However, whether the CAR can be used to predict complications in Crohn's disease (CD) patients after surgery has not yet been reported.

**Methods:**

A total of 534 CD patients undergoing surgery between 2016 and 2020 were enrolled. The risk factors of postoperative complications were assessed by univariate and multivariate analyses. The cutoff values and the accuracy of diagnosis for the CAR and postoperative CRP levels were examined with receiver operating characteristic (ROC) curves.

**Results:**

The rate of postoperative complications was 32.2%. The postoperative CAR (OR 13.200; 95% CI 6.501-26.803; *P* < 0.001) was a significant independent risk factor for complications. Compared with the CRP level on postoperative day 3, the CAR more accurately indicated postoperative complications in CD patients (AUC: 0.699 vs. 0.771; Youden index: 0.361 vs. 0.599). ROC curves showed that the cutoff value for the CAR was 3.25. Patients with a CAR ≥ 3.25 had more complications (*P* < 0.001), a longer postoperative stay (15.5 ± 0.6 d vs. 9.0 ± 0.2 d, *P* < 0.001), and more surgical site infections (48.2% vs. 5.7%, *P* < 0.001) than those with a CAR < 3.25.

**Conclusions:**

Compared to the CRP level, the CAR can more accurately predict postoperative complications and can act as a predictive marker in CD patients after surgery.

## 1. Introduction

Drug-induced remission can often be achieved in Crohn's disease (CD) patients, but as the disease progresses, patients eventually must undergo surgical treatment [1, 2]. The common postoperative complications in CD patients are postoperative bleeding, anastomotic leakage, abdominal abscess, intestinal obstruction, and short bowel syndrome, which can increase the treatment costs, prolong hospital stays, and reduce the long-term survival rate [3]. Therefore, the timely detection of postoperative outcomes is very important to improve the prognosis of CD patients.

Destruction of the intestinal structure leads to gradual worsening of the nutritional status of CD patients. Malnutrition and anemia contribute to an elevated incidence of postoperative complications [4, 5]. At present, a variety of predictors have been proposed to predict outcomes after surgery, such as the C-reactive protein (CRP), procalcitonin (PCT), platelet-to-lymphocyte ratio (PLR), neutrophil to lymphocyte ratio (NLR), body mass index (BMI), sarcopenia, and albumin (ALB) levels [6–10]. However, most predictors are assessed before surgery, and they cannot reflect inflammation caused by surgical stress. The systemic inflammatory response after surgery obviously affects postoperative outcomes [11]. Additionally, most CD patients will undergo some type of preoperative optimization, such as nutrition therapy before surgery. Thus, the PLR, NLR, BMI, sarcopenia, and other predictors will be improved before surgery, and these predictors are always only related to nutritional status but ignore the surgical stress.

Many studies have confirmed that CRP can be used as an inflammatory index to reflect the degree of trauma and inflammatory state [12]. The CRP level on postoperative day (POD) 3 or 4 was reported to be the best predictor of postoperative complications [13, 14]. In addition, the plasma level of ALB reflects not only the nutritional status of the body, but also inflammation from surgical trauma. Galata et al. [15] found that the preoperative ALB level was an independent predictor of major postoperative complications in CD patients after colorectal surgery. Ghoneima et al. [16] found that the preoperative CRP, haemoglobin (Hb), and ALB levels can act as predictors of septic complications in CD patients after surgery. Recently, some scholars have proposed that the CRP/ALB ratio (CAR) can predict postoperative complications in a timely manner in colorectal cancer, and its predictive value is better than that of CRP alone [17, 18]. Thus, the postoperative CAR including ALB and CRP not only reflects nutritional status, but also is associated with the systemic inflammatory response after surgery. However, few studies have assessed the role of the CAR in predicting postoperative complications in CD patients.

In the current study, we investigated the relationship between the CAR and postoperative outcomes in CD patients and compared the diagnostic accuracy of the CAR with that of the postoperative CRP level.

## 2. Methods and Materials

### 2.1. Patients

The clinical records of consecutive CD patients undergoing surgery were collected and retrospectively reviewed. The inclusion criteria included are as follows: (1) CD was diagnosed according to the European Crohn's and Colitis Organization (ECCO) guidelines [19], and (2) bowel resection was performed. The exclusion criteria included (1) incomplete laboratory data, (2) multiple organ failure, (3) closing of the ileostomy or colostomy, (4) emergency surgery, and (5) ALB infusion before the operation or within 2 days after the operation. This study was approved by the ethics committee of the School of Medicine, Zhejiang University.

### 2.2. Data Collection

The data included the patients' baseline characteristics (such as BMI and comorbidities), intraoperative data, and laboratory data (preoperative Hb level, preoperative ALB level, CRP level on POD1 and 3, ALB level on POD1, postoperative CAR, preoperative CRP level, preoperative erythrocyte sedimentation rate (ESR), preoperative white blood cell (WBC) count, preoperative red blood cell (RBC) count, preoperative platelet (PLT) count, and preoperative lymphocyte count) from the database. The CAR was defined as follows: CRP on POD1/ALB on POD1 × 100%.

### 2.3. Definition of Outcomes

Our study focused on the relationship between the CAR and postoperative complications in CD patients. Postoperative complications were defined as those that occurred within 30 days after surgery or before hospital discharge according to the Clavien-Dindo classification system [20]. Mild complications included Clavien-Dindo grade I or II complications, while major complications were Clavien-Dindo grades III-IV complications. The postoperative stay and surgical site infections (SSIs) were also collected from the database retrospectively. SSIs included surface incisional infections and deep space infections.

### 2.4. Statistical Analysis

SPSS 22.0 was used to analyze all data. Continuous data are reported as means ± standard deviations or medians (interquartile ranges), whereas categorical variables are described as numbers (percentages). Continuous data were analyzed using Student's *t*-tests, while categorical variables were analyzed by Pearson's *χ*^2^ test. The critical cutoff value for the CAR was calculated based on the ROC curve and Youden index. The potential independent risk factors for predicting postoperative outcomes were identified. Risk factors with a value of *P* < 0.1 were evaluated in the multivariate analysis. A *P* value <0.05 was considered statistically significant.

## 3. Results

### 3.1. Patients' Characteristics

Finally, 534 patients were enrolled in the study, and 120 patients were excluded according to the inclusion and exclusion criteria. A total of 299 (56.0%) patients underwent surgery for the first time. Some patients underwent preoperative optimization due to malnutrition or abdominal abscesses, including 158 (29.6%) CD patients who received exclusive enteral nutrition, 67 (12.5%) who received total parenteral nutrition, and 65 (12.2%) who received abscess drainage before surgery. In total, 172 (32.2%) patients experienced postoperative complications. Among those with postoperative complications, 135 (25.3%) patients had Clavien-Dindo grades I-II complications, and 97 (18.2%) patients had Clavien-Dindo grades III-IV complications. One hundred and three (19.3%) patients had SSIs, of which 58 (10.9%) were incisional SSIs, while 44 (8.2%) were organ/deep space SSIs (Table 1).

### 3.2. Assessment of Risk Factors Related to Postoperative Complications by Univariate and Multivariate Analyses

The baseline characteristics, intraoperative data, and preoperative laboratory data of patients with and without postoperative complications are described in Table 1. The potential independent risk factors that could predict postoperative complications were the postoperative CRP level, preoperative Hb, postoperative CAR, postoperative ESR, preoperative CRP level, operative time, estimated blood loss, BMI, preoperative ALB level, exclusive enteral nutrition, surgical history, and laparoscopic approach. Multivariate logistic regression analysis showed that the postoperative CAR (OR 13.200; 95% CI 6.501-26.803; *P* < 0.001), BMI (OR 0.432; 95% CI 0.261-0.713; *P* = 0.001), postoperative ESR (OR 1.774; 95% CI 1.021-3.084; *P* = 0.042), laparoscopic surgery (OR 0.496; 95% CI 0.267-0.923; *P* = 0.027), and exclusive enteral nutrition (OR 0.461; 95% CI 0.261-0.813; *P* = 0.007) were still independent risk factors for postoperative complications, as shown in Table 2.

### 3.3. Receiver Operating Characteristic (ROC) Curve Analysis of the CAR and the Optimal Cutoff Value for the Prediction of Surgical Outcomes

**Figure 1:**
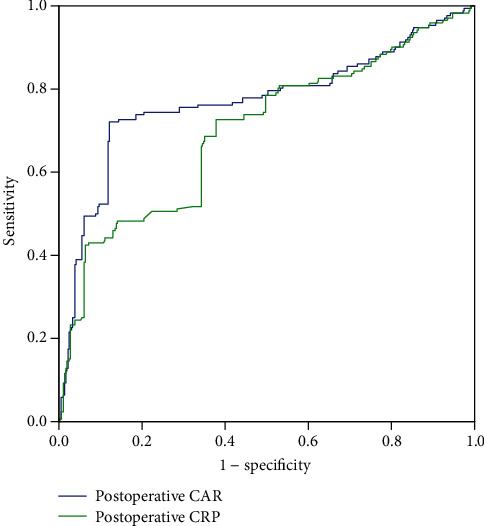
ROC curve showing postoperative CAR and CRP on POD3 levels predict postoperative complications. CAR: the C-reactive protein/albumin ratio; CRP: C-reactive protein.

This study determined the optimal critical cutoff value of the CAR, and the ROC curve of the CAR on POD1 and CRP level on POD3 was evaluated (as shown in Table 3 and Figure 1). The area under the curve (AUC), positive predictive value (PPV), negative predictive value (NPV), and cutoff point for CRP on POD3 were 0.699, 76.0%, 77.4%, and 125.8, respectively. However, the AUC, PPV, NPV, and cutoff point for CAR on POD1 were 0.707, 58.7%, 89.5%, and 3.25, respectively. Additionally, the Youden index of the CAR on POD1 was higher than that of the CRP on POD3 (0.482 vs. 0.361). Thus, the CAR can more accurately predict postoperative complications in CD patients than the CRP level.

### 3.4. Postoperative Complications of CD Patients with Low and High CAR Values

Patients were divided into two groups according to the cutoff value of the CAR (Table 4). The overall number of postoperative complications was 48 (13.1%) in the low-CAR group (CAR < 3.25) and 124 (73.8%) in the high-CAR group (CAR ≥ 3.25) (*P* < 0.001). The rate of mild postoperative complications was higher in the high-CAR group than in the low-CAR group (96 (57.1%) vs. 40 (10.9%), *P* < 0.001), including wound infections (46 (27.4%) vs. 12 (3.3%), *P* < 0.001), fever with a temperature > 38.5°C (25 (14.9%) vs. 8 (2.2%), *P* < 0.001), diarrhea (6 (3.6%) vs. 1 (0.3%), *P* = 0.003), and postoperative blood transfusion (10 (6.0%) vs. 3 (0.8%), *P* = 0.001). In addition, the number of major complications was also significantly higher in the high-CAR group (69 (41.1%) vs. 28 (7.7%), *P* < 0.001), including anastomotic leakage (25 (14.9%) vs. 5 (1.4%), *P* < 0.001). Furthermore, patients with a CAR ≥ 3.25 had a significantly longer postoperative stay (15.5 ± 0.6 vs. 9.0 ± 0.2, *P* < 0.001) (Table 4). With regard to SSIs, incisional SSIs occurred in 58 (10.9%) patients, while organ/deep space SSIs occurred in 44 (8.2%) patients. The rates of incisional and organ/deep space SSIs were significantly higher in the high-CAR group (46 (27.4%) vs. 12 (3.3%), *P* < 0.001; 35 (20.8%) vs. 9 (2.5%), *P* < 0.001, respectively).

## 4. Discussion

This study showed that the CAR was a reliable and accurate indicator of postoperative outcomes in CD patients. The CAR on POD1 was a better predictor of postoperative complications than the CRP on POD3. Moreover, CD patients with a CAR ≥ 3.25 had more postoperative complications, longer postoperative stays, and more SSIs.

With the progression of the disease, many CD patients eventually require surgery [21, 22]. Costa et al. [1] reported that 70% of CD patients eventually needed surgical intervention, and postoperative complications were very common. Therefore, it is very important to identify an accurate method to predict the risk of postoperative complications in CD patients to guide early clinical interventions, improve the outcome, and reduce complications.

Many studies have confirmed that the inflammatory response after surgery is a risk factor for postoperative complications [23], including factors such as CRP, serum amyloid A, and IL-6 [24–26]. ALB is also considered to be an indicator of short-term and long-term postoperative outcomes in CD patients [27, 28]. In addition, some newer predictive scores depend on inflammation, including the modified Glasgow Outcome Score (MGPS), NLR, and CAR [29–32], and can also be used to predict complications in patients undergoing colorectal surgery. Haruki et al. [33] suggested that the CAR was an independent risk factor of poor long-term outcomes of pancreatic resection. A meta-analysis by Wang et al. [34] showed that in patients with colorectal cancer, an increased CAR was associated with a poor outcome. They suggested that the CAR was a predictive factor that could be used to classify colorectal patients according to risk. Our results also demonstrated that the CAR could predict postoperative complications in CD patients.

CRP is an important index for evaluating the activity of CD, and it can also predict postoperative complications [35]. However, the postoperative complications in CD patients are not only related to inflammation but also closely related to nutritional status, which is reflected in the ALB level. Low ALB levels negatively affect the prediction of patient outcomes [36]. Therefore, for patients with CD, it is far from sufficient to use only the CRP level to predict their postoperative outcome, as indicated in a study by Easton and Balogh [37], who showed some drawbacks of using the CRP level. Hence, the CAR, which incorporates both the CRP and ALB levels, can predict the outcome of CD patients after bowel resection, and its accuracy is better than that of the CRP level according to the current study. To our knowledge, this study is the first to explore and compare the predictive ability of the CAR for postoperative complications in CD patients.

This result is not unexpected because the CRP level represents the degree of inflammation in patients, which is a risk factor for poor wound healing and infection and is related to a poor outcome [38–40]. The ALB level indicates the nutritional status of patients. Hypoproteinemia is associated with inflammation or previous malnutrition [41], which can lead to muscle atrophy and respiratory and immune dysfunction, thus prolonging the postoperative recovery time and increasing the incidence of postoperative complications in CD patients [42]. Acute stress can cause damage to vascular endothelial function, allowing ALB to move into the interstitial space, which causes tissue edema and insufficient perfusion, leading to a series of complications. Therefore, a higher CRP level and lower ALB level result in a higher CAR, which increases the likelihood that patients will develop postoperative complications.

Patients with a very high postoperative CAR should be intensively monitored for early detection of complications. Thus, these results have important implications for clinicians to optimally implement prophylactic strategies during the early postoperative period to improve outcomes in CD patients after bowel resection, including ALB infusion, prolongation of antibiotic administration, and other examinations to detect complications. Surgeons are advised to be aware of the CAR during the early postoperative period, even for patients with normal preoperative ALB and CRP levels.

Despite our interesting findings, there are still several limitations of the current study. First, this single-center study included a homogeneous cohort of patients who underwent surgery with a fixed surgical team. Second, the retrospective nature of this study meant that selection bias could have occurred. Therefore, prospective multicenter studies are warranted to confirm role of the CAR in predicting the short-term and long-term prognoses of CD patients after surgery.

## 5. Conclusions

Our results showed that the CAR, a new and feasible tool, has a significant correlation with postoperative complications and can serve as a predictive marker in CD patients undergoing bowel resection. Compared with the CRP level, the CAR is more accurate for the prediction of postoperative complications and could help clinicians evaluate the precise risk level and nutritional status of patients earlier. When patients have a CAR greater than 3.25, clinicians should be vigilant, continuously monitor the occurrence of postoperative complications, and provide timely interventions to improve their outcome.

## Figures and Tables

**Table 1:** Characteristic of participants and univariate analysis of risk factors associated with postoperative complications.

Characteristics	All (534)	With complications (172)	Without complications (362)	*P* value
Age	34.9 ± 0.5	35.0 ± 0.8	34.9 ± 0.6	0.928
Men	375 (70.2)	119 (69.2)	256 (70.7)	0.717
BMI	18.7 ± 0.1	18.1 ± 0.2	19.0 ± 0.1	<0.001
Preoperative Hb	11.9 ± 0.1	11.7 ± 0.2	12.1 ± 0.1	0.054
Preoperative ALB	36.5 ± 0.2	35.5 ± 0.5	37.0 ± 0.2	0.001
Postoperative CRP	82.2 ± 2.1	105.8 ± 4.1	71.0 ± 2.1	<0.001
Postoperative CAR	2.7 ± 0.1	3.7 ± 0.2	2.3 ± 0.1	<0.001
Preoperative CRP	16.7 ± 1.4	25.4 ± 3.6	12.5 ± 1.0	<0.001
Preoperative ESR	19.3 ± 0.7	17.5 ± 1.2	20.1 ± 0.9	0.086
Preoperative WBC	6.3 ± 0.1	6.6 ± 0.2	6.2 ± 0.1	0.120
Preoperative RBC	4.22 ± 0.03	4.16 ± 0.05	4.25 ± 0.03	0.133
Preoperative PLT	250.3 ± 3.6	253.3 ± 6.3	249.0 ± 4.3	0.572
Preoperative lymphocyte	1.10 ± 0.02	1.06 ± 0.04	1.12 ± 0.02	0.176
Current smoking	20 (3.7)	7 (4.1)	13 (3.6)	0.785
Disease duration before surgery	53.1 ± 2.1	55.4 ± 3.8	52.0 ± 2.6	0.457
Preoperative optimization				
Exclusive enteral nutrition	158 (29.6)	37 (21.5)	121 (33.4)	0.005
Total parenteral nutrition	67 (12.5)	17 (9.9)	50 (13.8)	0.200
Abscesses drainage	65 (12.2)	20 (11.6)	45 (12.4)	0.791
Montreal classification				
Age, y				
A1 (≤16)	3 (0.6)	2 (1.2)	1 (0.3)	0.224
A2 (17-40)	396 (74.2)	127 (73.8)	269 (74.3)	0.907
A3 (>40)	135 (25.3)	43 (25.0)	92 (25.4)	0.918
Location				
L1 (ileal)	147 (27.5)	55 (32.0)	92 (25.4)	0.113
L2 (colonic)	76 (14.2)	24 (14.0)	52 (14.4)	0.899
L3 (ileocolonic)	303 (56.7)	104 (60.5)	199 (55.0)	0.231
L4 (upper gastrointestinal)	44 (8.2)	15 (8.7)	29 (8.0)	0.780
Behavior				
B1 (inflammatory/failure of medical therapy)	35 (6.6)	9 (5.2)	26 (7.2)	0.395
B2 (stricturing)	348 (65.2)	109 (63.3)	239 (66.0)	0.418
B3 (penetrating)	199 (37.3)	72 (41.9)	127 (35.1)	0.130
Perianal disease	150 (28.1)	43 (25.0)	107 (29.6)	0.273
Operative time	203.3 ± 2.7	225.2 ± 5.2	192.9 ± 3.0	<0.001
ASA ≥ 3	45 (8.4)	14 (8.1)	31 (8.6)	0.869
First time operated	299 (56.0)	83 (48.3)	216 (59.7)	0.013
Laparoscopic surgery	264 (49.4)	50 (29.0)	214 (59.1)	<0.001
Conversion	101 (18.9)	36 (20.9)	65 (18.0)	0.412
Estimated blood loss	87.0 ± 3.5	122.3 ± 8.3	70.2 ± 2.9	<0.001
Preoperative treatment				
Azathioprine	111 (20.8)	33 (19.2)	78 (21.5)	0.530
Infliximab	115 (21.5)	43 (25.0)	72 (19.9)	0.179
5-ASA	144 (27.0)	45 (26.2)	99 (27.3)	0.773
Corticosteroids	25 (4.7)	5 (2.9)	20 (5.5)	0.181
Others	117 (21.9)	42 (24.4)	75 (20.7)	0.334

BMI: body mass index; Hb: haemoglobin; ALB: albumin; CRP: C-reactive protein; ESR: erythrocyte sedimentation rate; WBC: white blood cell; RBC: red blood cell; PLT: platelet; CAR: the CRP/ALB ratio.

**Table 2:** Multivariate analysis of factors associated with postoperative complications.

Characteristics	Multivariate		
	*P* value	OR	95% CI
BMI	0.001	0.432	0.261-0.713
Preoperative Hb	0.902	0.966	0.562-1.662
Preoperative ALB	0.267	0.670	0.330-1.358
Preoperative CRP	0.906	1.033	0.559-1.674
Postoperative CRP	0.348	1.443	0.670-3.106
Postoperative CAR	<0.001	12.630	6.153-25.925
Postoperative ESR	0.042	1.774	1.021-3.084
Operative time	0.174	1.455	0.847-2.499
First time operated	0.847	0.951	0.568-1.592
Laparoscopic surgery	0.027	0.496	0.267-0.923
Estimated blood loss	0.665	0.877	0.485-1.587
Exclusive enteral nutrition	0.007	0.461	0.261-0.813

BMI: body mass index; Hb: haemoglobin; ALB: albumin; CRP: C-reactive protein; CAR: the CRP/ALB ratio.

**Table 3:** ROC curve showing postoperative CAR levels on POD1 and CRP on POD3 levels predict postoperative complications.

Characteristics	Postoperative CRP	Postoperative CAR
Cutoff point	125.8	3.25
AUC	0.699	0.707
Sensitivity	0.424	0.587
Specificity	0.936	0.895
Positive predictive value	0.760	0.738
Negative predictive value	0.774	0.869
Youden index	0.361	0.482

CRP: C-reactive protein; CAR: the CRP/ALB ratio.

**Table 4:** Comparison of postoperative complications for bowel resection in CD patients according to postoperative CAR.

Characteristics	All (*n* = 534)	CAR < 3.25 (*n* = 366)	CCR ≥ 3.25 (*n* = 168)	*P* value
Postoperative complications	172 (32.2)	48 (13.1)	124 (73.8)	<0.001
Mild complications (grades I to II)	135 (25.3)	40 (10.9)	96 (57.1)	<0.001
Wound infection	57 (10.7)	12 (3.3)	46 (27.4)	<0.001
Fever > 38.5°C after surgery	33 (6.2)	8 (2.2)	25 (14.9)	<0.001
Diarrhea	7 (1.3)	1 (0.3)	6 (3.6)	0.003
Early postoperative bowel obstruction	21 (3.9)	13 (3.6)	8 (4.8)	0.504
Postoperative blood transfusions	13 (2.4)	3 (0.8)	10 (6.0)	0.001
Line sepsis	3 (0.5)	2 (0.5)	1 (0.6)	0.944
Hyperglycemia	1 (0.2)	1 (0.3)	0 (0)	0.384
Major complications (grade III to IV)	97 (18.2)	28 (7.7)	69 (41.1)	<0.001
Gastrointestinal bleeding	13 (2.4)	10 (2.7)	3 (1.8)	0.498
Anastomotic leakage	30 (5.6)	5 (1.4)	25 (14.9)	<0.001
Abdominopelvic collection	6 (1.1)	2 (0.5)	4 (2.4)	0.075
Pleural effusion	4 (0.7)	1 (0.3)	3 (1.8)	0.073
Intra-abdominal abscess	15 (2.8)	4 (1.1)	11 (6.5)	0.001
Stoma complications	17 (3.2)	3 (0.8)	14 (8.3)	<0.001
Septic shock	6 (1.1)	2 (0.5)	4 (2.4)	0.075
Sepsis	5 (0.9)	1 (0.3)	4 (2.4)	0.025
Kidney failure	1 (0.2)	0 (0)	1 (0.6)	0.128
Grade V	0 (0)	0 (0)	0 (0)	-
Postoperative stay^∗^, days	11.1 ± 0.3	9.0 ± 0.2	15.5 ± 0.6	<0.001
SSIs	103 (19.3)	21 (5.7)	81 (48.2)	<0.001
Incisional SSI	58 (10.9)	12 (3.3)	46 (27.4)	<0.001
Organ/space SSI	44 (8.2)	9 (2.5)	35 (20.8)	<0.001

CD: Crohn's disease; CAR: the CRP/ALB ratio; SSIs: surgical site infections.

## Data Availability

The datasets were available from the corresponding author (gxlmed@zju.edu.cn).

## Abbreviations

Hb: Haemoglobin
ALB: Albumin
CRP: C-reactive protein
PLT: Platelet
CAR: The CRP/ALB ratio
CD: Crohn's disease
PLR: Platelet-to-lymphocyte ratio
NLR: Neutrophil to lymphocyte ratio
BMI: Body mass index
ESR: Erythrocyte sedimentation rate
WBC: White blood cell
RBC: Red blood cell
SSIs: Surgical site infections.
